# Differential effects of climate and species interactions on range limits at a hybrid zone: potential direct and indirect impacts of climate change

**DOI:** 10.1002/ece3.1774

**Published:** 2015-10-19

**Authors:** Michael A. McQuillan, Amber M. Rice

**Affiliations:** ^1^ Department of Biological SciencesLehigh University111 Research DriveBethlehemPennsylvania18015

**Keywords:** Biotic interactions, chickadee, contact zone, ecological niche model, hybridization, MAXENT, *Poecile atricapillus*, *Poecile carolinensis*, range expansion, species distribution model

## Abstract

The relative contributions of climate versus interspecific interactions in shaping species distributions have important implications for closely related species at contact zones. When hybridization occurs within a contact zone, these factors regulate hybrid zone location and movement. While a hybrid zone's position may depend on both climate and interactions between the hybridizing species, little is known about how these factors interact to affect hybrid zone dynamics. Here, we utilize SDM (species distribution modeling) both to characterize the factors affecting the current location of a moving North American avian hybrid zone and to predict potential direct and indirect effects of climate change on future distributions. We focus on two passerine species that hybridize where their ranges meet, the Black‐capped (*Poecile atricapillus*) and Carolina (*P. carolinensis*) chickadee. Our contemporary climate models predict the occurrence of climatically suitable habitat extending beyond the hybrid zone for *P. atricapillus* only, suggesting that interspecific interactions primarily regulate this range boundary in *P. atricapillus*, while climatic factors regulate *P. carolinensis*. Year 2050 climate models predict a drastic northward shift in suitable habitat for *P. carolinensis*. Because of the greater importance of interspecific interactions for regulating the southern range limit of *P. atricapillus*, these climate‐mediated shifts in the distribution of *P. carolinensis* may indirectly lead to a range retraction in *P. atricapillus*. Together, our results highlight the ways climate change can both directly and indirectly affect species distributions and hybrid zone location. In addition, our study lends support to the longstanding hypothesis that abiotic factors regulate species' poleward range limits, while biotic factors shape equatorial range limits.

## Introduction

A major focus in the fields of ecology and evolutionary biology is to tease apart the relative contributions of abiotic and biotic factors in shaping species distributions (MacArthur 1972; Sexton et al. 2009). The effects of abiotic factors, such as climate, on species distributions are well characterized and especially important to examine given the rapid rate of global climate change (Hutchinson 1957; Parmesan 2006). Biotic factors, such as interspecific interactions, also play a major role in determining species distributions (Connell 1961; Bullock et al. 2000; Case et al. 2005; Cunningham et al. 2009). When species' ranges are adjacent to one another (i.e., parapatric), access to climatically suitable habitat could be limited due to negative interspecific interactions with competitors, predators, or mates (Case and Taper 2000; Anderson et al. 2002; Arif et al. 2007; Gröning and Hochkirch 2008). In such cases, there is often a mismatch between a species' realized distribution – where it actually occurs – and its potential distribution, which includes all habitat areas that are climatically suitable. Yet, even when biotic factors outweigh abiotic factors in shaping a species' distribution, climatic factors may have significant indirect impacts if such factors strongly influence the distribution of an interacting species (Thomas 2010). Such indirect impacts of climate are likely to become increasingly important as climate change‐mediated range changes bring previously isolated species into contact.

The study of closely related species that form contact zones at parapatric range edges can provide insight into the factors determining range limits (Cicero 2004; Swenson 2006). For example, interspecific competition within a contact zone can cause one taxon to be competitively excluded from colonizing novel areas (Case et al. 2005), regardless of the presence of climatically suitable habitats. If competitive ability is asymmetric between species, the distribution of the superior competitor may be determined by abiotic factors, while the inferior competitor may be limited to areas not climatically suitable for the superior competitor. Indeed, empirical evidence supports a role for interspecific competition in shaping species distributions (Arif et al. 2007; Jankowski et al. 2010; Gutiérrez et al. 2014).

In a similar fashion, the production of unfit hybrid offspring within a contact zone can limit range expansion by one or both parental species into climatically suitable habitats (Goldberg and Lande 2006). Such naturally occurring hybrid zones are particularly interesting examples of range boundaries, due to the interplay of intrinsic and extrinsic forces that determine their position in space and time. Clinal hybrid zones, where phenotypic and/or genetic character states are distributed in a clinal fashion as one moves across the zone, are common in nature and can be maintained by a number of selective regimes (Teeter et al. 2008; Delmore et al. 2013; Smith et al. 2013). Such a hybrid zone is often maintained due to a balance between dispersal of parental forms into the zone and strong intrinsic selection against hybrids (i.e., a tension zone) (Barton and Hewitt 1985, 1989; Barton 2001). The location of such zones may therefore be dependent on the densities of the hybridizing species. Tension zones can move across the landscape and may settle in troughs of low population density. Alternatively, selection against hybrids can be extrinsic and depend on abiotic environmental factors (Kruuk et al. 1999). Under this scenario, selection may favor one parental type at each end of an environmental gradient, and the hybrid zone might be located at an ecotone or environmental transition (Endler 1977). When these criteria are met, multiple independent hybrid zones can even cluster in these areas (Moore and Price 1993; Swenson and Howard 2005; Swenson 2006). Finally, a clinal hybrid zone's position may be maintained because hybrid individuals experience a selective advantage in intermediate environments (i.e., bounded hybrid superiority) (Moore 1977). Importantly, both intrinsic and extrinsic sources of selection may act simultaneously in natural hybrid zones, and therefore, some zones may share characteristics of multiple of these described models (e.g., Bert and Arnold 1995; Delmore et al. 2013). In such cases, interactions between biotic and abiotic factors may be responsible for determining range limits.

**Figure 1:**
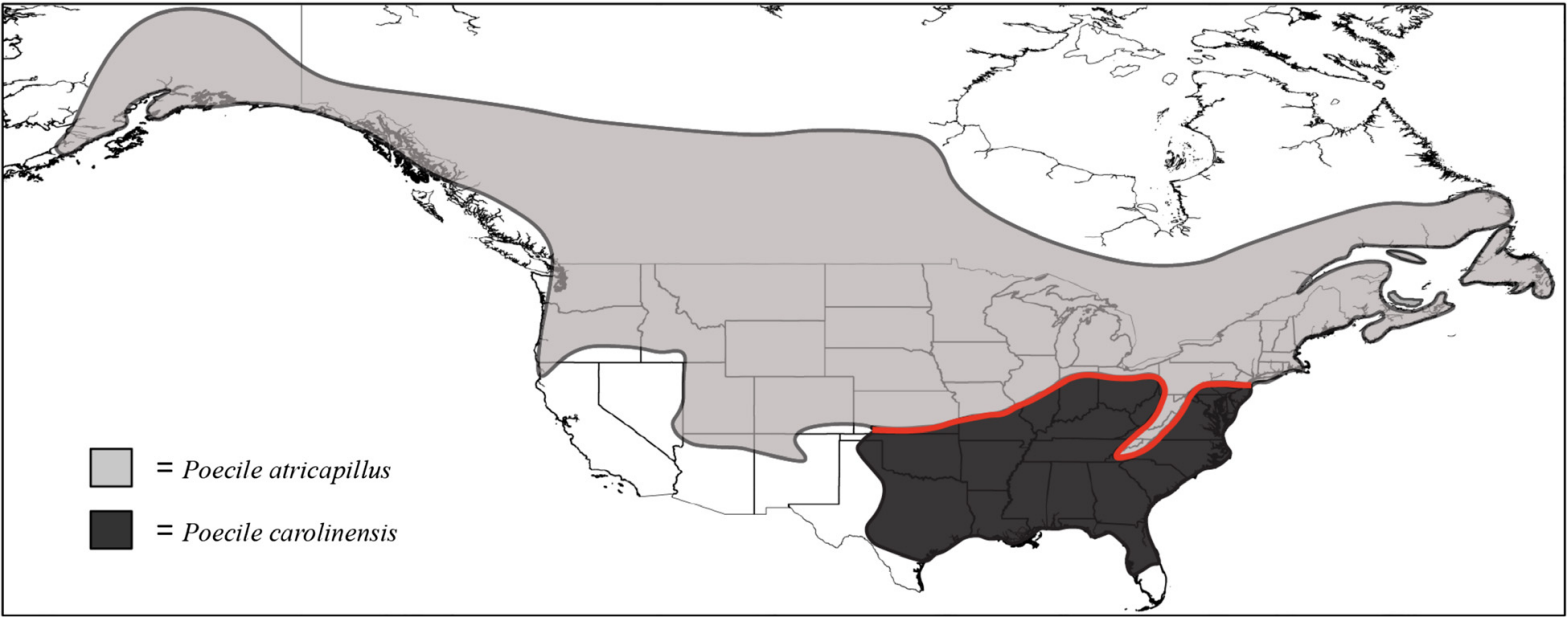
Geographical distribution of Black‐capped chickadees (*Poecile atricapillus*) and Carolina chickadees (*Poecile carolinensis*). Approximate location of hybrid zone drawn as red line. Species distributions and hybrid zone location based on Taylor et al. (2014a).

Here, we examine two species of hybridizing North American passerine birds, the Black‐capped (*Poecile atricapillus*) and Carolina (*Poecile carolinensis*) chickadee, to elucidate the relative importance of abiotic and biotic factors in shaping species distributions along a natural contact zone. Chickadees are an ideal system in this regard, for a number of reasons. First, these geographically widespread species hybridize along a narrow east‐to‐west band stretching from Kansas to New Jersey (Fig. 1). The hybrid zone is not associated with any known physical barrier to dispersal. Multiple lines of evidence suggest strong intrinsic selection is acting against hybrids, in the form of lower hatching success (Bronson et al. 2003b, 2005). Further, recent genomic work examining a portion of the hybrid zone in Pennsylvania suggests that introgression across species boundaries is limited, and thus, the chickadee hybrid zone has been hypothesized to be a tension zone (Taylor et al. 2014b). Second, the hybrid zone is moving rapidly northward at a rate of approximately 10 km/decade, with *P. carolinensis* moving into territory historically occupied by *P. atricapillus* and displacing them (Bronson et al. 2003a; Reudink et al. 2007; Taylor et al. 2014a). This northward movement has been linked to past climate change and correlates with warming winter temperatures (Taylor et al. 2014a). Finally, behavioral work suggests that *P. carolinensis* males are competitively dominant over *P. atricapillus* males (Bronson et al. 2003a). With evidence suggesting roles for both biotic (interspecific hybridization, competition) and abiotic (climate) factors underlying distribution patterns in these species, the chickadee system is ideal for evaluating the relative importance of each for determining range limits.

In this study, we used SDM (species distribution modeling) to address two goals. First, by characterizing the mismatch between potential and realized distributions along a naturally occurring contact zone, we sought to determine the relative importance of biotic and abiotic factors in shaping the geographic ranges of these two hybridizing bird species. Second, using recent climate change projections, we assess the potential direct and indirect effects of future climate change on the distribution patterns and hybrid zone movement dynamics in these species.

## Materials and Methods

We can gain insight into the relative importance of abiotic and biotic factors for determining species distribution patterns by measuring the extent of suitable habitat left unoccupied by a species. One method of characterizing this mismatch between a species' potential and realized distribution is SDM (Swenson 2008; Elith and Leathwick 2009; Franklin 2009). Correlative species distribution models characterize a species' niche by extracting environmental predictor variables (climate, elevation, soil type, etc.) from locations where the species is known to occur. This information, in conjunction with a statistical model, is used to determine the probability that the species will occur along each environmental axis; these occurrence probabilities are then projected onto a geographic study area. When used with climate variables, SDM can reveal the potential, climatically suitable niche of a species. This potential distribution can then be compared to the species' realized distribution. Once the potential distribution of a species has been modeled under current climate conditions, further analyses can project suitable habitat areas under a variety of predicted climate change scenarios (Hijmans and Graham 2006). Such analyses can inform biologists about potential species range shifts in the face of rapid environmental change (Engler et al. 2013; Wisz et al. 2013).

### Data acquisition

We extracted climate data from the WorldClim database (worldclim.org, Hijmans et al. 2005) at a 2.5‐arc‐minute resolution (~5 km^2^). We used a total of 19 bioclimatic variables plus altitude for distribution modeling (Table 1). These bioclimatic variables represent annual trends in temperature, precipitation, extreme climatic factors, and seasonality. We downloaded climate data for two time periods: contemporary conditions (climate averages for the years 1950–2000) and future conditions predicted for the year 2050 (averages for years 2041–2060). Once downloaded, we cropped the climate layers and formatted them to include the majority of North America, between latitudes 23.94N and 71.88N and between longitudes 49.42W and 168.35W. We chose this spatial extent because it contains the complete geographic ranges of both species.

**Table 1:** Climate variables used for the three types of distribution models

	Full models	Reduced model *Poecile atricapillus*	Reduced model *Poecile carolinensis*	Uncorrelated models
Annual Mean Temperature (Bio1)	✔	✔	✔	✔
Mean Diurnal Range in Temperature (Bio2)	✔	✔		
Isothermality (Bio3)	✔			
Temperature Seasonality (Bio4)	✔			
Max Temperature of Warmest Month (Bio5)	✔	✔		
Min Temperature of Coldest Month (Bio6)	✔			
Temperature Annual Range (Bio7)	✔			✔
Mean Temperature of Wettest Quarter (Bio8)	✔			✔
Mean Temperature of Driest Quarter (Bio9)	✔			
Mean Temperature of Warmest Quarter (Bio10)	✔		✔	
Mean Temperature of Coldest Quarter (Bio11)	✔	✔		
Annual Precipitation (Bio12)	✔		✔	
Precipitation of Wettest Month (Bio13)	✔			
Precipitation of Driest Month (Bio14)	✔		✔	
Precipitation Seasonality (Bio15)	✔			
Precipitation of Wettest Quarter (Bio16)	✔			✔
Precipitation of Driest Quarter (Bio17)	✔	✔	✔	✔
Precipitation of Warmest Quarter (Bio18)	✔			
Precipitation of Coldest Quarter (Bio19)	✔			
Altitude	✔			

We obtained species occurrence locations from eBird, a publically available citizen science database (ebird.org, *n* = 12,947), and from geo‐referenced museum specimens downloaded from the Global Biodiversity Information Facility (gbif.org, *n* = 324). *Poecile atricapillus* and *P. carolinensis* are permanent residents throughout their respective ranges, but we only used occurrence records taken during the breeding season (May and June) for distribution modeling. We limited our dataset to records from the years 1950 to 2000 in order to match with the timescale of available climate data (see above). Because presence‐only SDM methods assume unbiased, random sampling (Phillips et al. 2009; Yackulic et al. 2013), we took multiple measures to correct for any sampling bias in our occurrence dataset. First, because species identification may not be accurate within or near the contact zone (Kroodsma et al. 1995; Curry et al. 2007), we ensured that presence records represented pure‐species individuals by filtering our dataset as follows. We split our geographic study area into 0.17° (~20 km^2^) grid cells. If a grid cell contained presence records from both species, that location was considered to be within the contact zone and those records were discarded. Including records from both species in these locales did not significantly affect the models (data not shown). Second, to reduce spatial bias and spatial autocorrelation in our occurrence records, we further filtered the dataset by reducing multiple occurrence records to a single record within a given Euclidian distance (Veloz 2009; Boria et al. 2014). Specifically, we employed a graduated filtering method based on climate heterogeneity using the program SDMtoolbox (Brown 2014). With this method, the first three principal components were calculated for all input climate data and then used to estimate climate heterogeneity. This splits the study area into areas of high and low climate heterogeneity. In areas where climate is highly heterogeneous, occurrence records were filtered at a resolution of 5 km. In contrast, in areas of low climate heterogeneity, occurrence records were filtered at a resolution of 25 km. Finally, because our occurrence records still appeared to be spatially biased toward some areas, we took this spatially rarefied dataset and used it to generate a Gaussian kernel density map that up‐weights occurrence records with fewer neighbors in geographic space, using the program SDMtoolbox (Brown 2014). The output map from this analysis was used as a bias grid in all modeling runs (Elith et al. 2011; Fourcade et al. 2014). Even after taking these measures, completely removing all sources of bias from citizen science databases is difficult. However, for geographically widespread species such as chickadees, eBird occurrences offer certain advantages that planned, systematic sampling surveys cannot. Our occurrence dataset is more geographically extensive than any single random sampling effort and thus likely captures a more representative set of environmental conditions experienced by the focal species across the entire landscape. After applying these filtering constraints and removing duplicate records, 2345 Black‐capped (*P. atricapillus*) and 490 Carolina (*P. carolinensis*) chickadee records were used for constructing the final species distribution models.

### Species distribution modeling

If abiotic factors, such as climate, are important for shaping species distributions, then the potential, climatically suitable distribution of a species should closely match that species' realized distribution. Conversely, if biotic factors, such as negative interspecific interactions, are more important for determining range limits, then a species' potential distribution should extend far beyond its realized range. Applying these predictions to hybrid zones, if a zone's location is determined by the outcomes of interspecific interactions (biotic factors), then the potential distribution of both hybridizing species should extend beyond the hybrid zone and into the actual range of the other species. If, however, a hybrid zone's location is determined by abiotic factors, such as climate, then the potential range of each species should not extend beyond the contact zone between them.

To determine the potential, climatically suitable distribution for both *P. atricapillus* and *P. carolinensis*, we used the SDM program MAXENT (ver. 3.3.3k, Phillips et al. 2006). We chose MAXENT as our modeling algorithm because it performs relatively well compared with other modeling methods, and it requires species presence data only (Elith et al. 2006). MAXENT extracts climatic data from each species occurrence location and uses this information in conjunction with random background sampling to estimate the distribution of suitable habitat conditions across geographic space. Suitable habitat areas are estimated according to the principle of maximum entropy, where the most likely distribution is the one that is the most spread out, or closest to uniform, subject to constraints imposed by the chosen climate variables (Phillips et al. 2006; Elith et al. 2011). We used the logistic output in MAXENT, which assigns each grid cell of the study area a value between 0 (unsuitable habitat) and 1 (fully suitable habitat). The model output grids were converted to heat maps with warmer colors indicating higher predicted habitat suitability, and visualized in ArcMap (ver. 10.2.2; Environmental Systems Research Institute [ESRI], Redlands, CA, USA).

We used the default MAXENT settings to model potential distributions for both species. We ran each model 10 times using a 10‐fold cross‐validation procedure, which splits the species occurrence data into 10 independent subsets, each with the same number of occurrence points (Elith et al. 2011). Nine subsets are used to train the model, and one subset is used to test the model. By repeating this procedure with all possible combinations of subsets, the true predictive power of the model can be evaluated. We report the average of these 10 runs as our modeling results, and also used this average for all further analyses, except as noted below. Furthermore, we used the AUC (area under curve) of the ROC (receiver operating characteristic) statistic to evaluate the fit of the models to the test data (Fielding and Bell 1997; Araújo et al. 2005).

Because species distribution models constructed in MAXENT can be sensitive to the specific climate variables chosen (Rödder and Lötters 2009), we ran several independent distribution models consisting of different subsets of variables. First, we ran one model for each species using the full set of 19 bioclimatic variables plus altitude (full models). Next, we used the results from the full models and ran another set of models using only the top five contributing variables to construction of the full models (reduced models). This method of model simplification resulted in one reduced model for each species based on different subsets of variables, as the relative contributions of the climate variables to the full models differed between the species. Finally, because bioclimatic variables are often highly correlated, we selected only those variables from the original set that were relatively uncorrelated with one another. To do this, we randomly selected 10,000 background points from the geographic study area and extracted the climate data from each point. We then determined the degree of correlation for each variable pair by calculating pairwise Pearson's correlation coefficients (*r*) (Table S1). Because annual mean temperature (bio1) contributed most to the full models for both *P. atricapillus* and *P. carolinensis* (see Results), we first dropped all climate variables that were correlated with this variable (*r* > 0.7). We then used the pairwise correlation matrix to randomly drop one climate variable from each pair of the remaining variables whenever the pairwise *r* > 0.7, until we were left with a set of relatively uncorrelated variables. We used this set of variables to run one additional model for each species (uncorrelated models). Table 1 lists the climate variables used for each of the three model types (Full, Reduced, and uncorrelated models).

To assess similarity between models run using different sets of predictor variables, we calculated Pearson's correlation coefficient (*r*) for the output logistic values between each pair of models using the program SDMtoolbox (Brown 2014). We conducted the same correlational analysis to compare modeling output under a variety of predicted future climate scenarios (see below; Tables S2, S3). Additionally, in order to determine which climate variable most influences model prediction across the study area, we employed a limiting factor analysis as described in Elith et al. (2010). This analysis returns a map of the geographic study area and indicates the climate variable that most influences model prediction at each individual grid cell. At each grid cell, the value of each climate variable is changed in turn, and the variable which when changed, leads to the largest increase in predicted probability of occurrence, is considered the limiting climatic variable. Because the limiting factor analysis could not be performed on the average output of our 10 cross‐validated runs, we ran one additional full model for each species where all of the occurrence data were used for training the model. We performed the limiting factor analysis on the results from these full models (see Elith et al. 2010, appendix S3, for further information and code used to implement this analysis in MAXENT).

Our second goal was to assess the potential direct and indirect effects of climate change on the species distributions and hybrid zone movement. To do this, we projected the MAXENT results from the full models of both species onto climate conditions for the year 2050. We used climate data based on two different climate change scenarios derived from the Intergovernmental Panel on Climate Change's Fifth Assessment Report (IPCC, 2013). Specifically, we used two GCMs (general circulation models), to account for uncertainty in climate change predictions: the HadGEM2‐ES (Hadley Global Environment Model 2 – Earth System) and CCSM 4.0 (Community Climate System Model) GCMs (Collins et al. 2011; Jones et al. 2011). For each of these two scenarios, we employed two different RCPs (representative concentration pathways), which describe greenhouse gas concentration trajectories for the years to come. The two concentration pathways we used represent a relatively optimistic future (RCP4.5), where greenhouse gas emissions begin to decline after the year 2040, and a relatively pessimistic future (RCP8.5), where emissions rise consistently throughout the next century (Meinshausen et al. 2011). Climate layers for future climate scenarios were downloaded from the WorldClim database (worldclim.org).

In order to quantify species distribution changes under future climate change conditions, we compared SDMs constructed for contemporary conditions with those projected onto predicted conditions for the year 2050. To do this, we first converted our full model SDMs for contemporary and future conditions from a continuous logistic output to a binary classification of either suitable or unsuitable area, using a threshold approach. Specifically, we chose the ‘maximum training sensitivity plus specificity’ as specified by MAXENT as our threshold, where grid cells with values greater than the threshold were classified as suitable habitat and grid cells with values lower than the threshold were classified as unsuitable. We chose this particular threshold because it has been shown to give a relatively accurate presence/absence prediction compared with other thresholds (Liu et al. 2005) (see Fig. S1 for binary threshold SDMs). Once we converted the SDMs to a binary format, we calculated the area (km^2^) of range expansion, range contraction, and no distribution change between contemporary and future SDMs and visualized these results using the program SDMtoolbox (Brown 2014). Because the different future climate change scenarios predicted similar areas of habitat suitability (see Results), we only conducted this analysis using the HADGEM2‐ES GCM under RCP 4.5.

## Results

**Figure 2:**
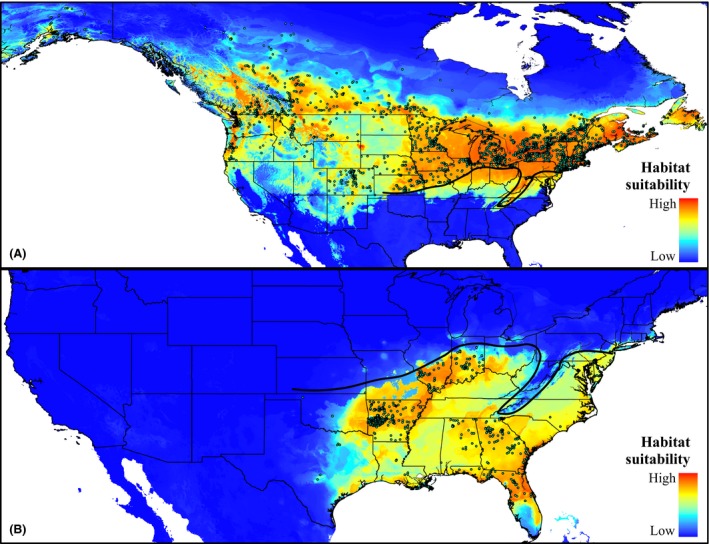
MAXENT species distribution models (full models) for *Poecile atricapillus* and *Poecile carolinensis* under contemporary conditions. (A) Black‐capped (*P*. atricapillus) chickadee potential distribution. (B) Carolina (*P. carolinensis*) chickadee potential distribution. Warmer colors indicate higher predicted habitat suitability. Species occurrence points used for modeling shown as blue circles. Approximate location of hybrid zone drawn as heavy black line (based on Taylor et al. 2014a).

Our SDM results show a large mismatch between the potential and realized distribution along the parapatric range edge (contact zone) for *P. atricapillus*, but not for *P. carolinensis* (Figs. 2, S2). The *P. atricapillus* potential, climatically suitable distribution extends south beyond the hybrid zone, and into the actual range of *P. carolinensis* (Figs. 2A, S2a). This mismatch was not seen in the *P. carolinensis* distribution models; the potential distribution closely matches the species' actual, realized distribution (Figs. 2B, S2b).

All three models run using different sets of climatic predictor variables showed a high degree of similarity in the habitat areas that were predicted to be climatically suitable. All pairwise Pearson's correlation coefficients (*r*) between the three model types (full models, reduced models, uncorrelated models) for both species were >0.96 (Table 2). For this reason, we present here only the results from the full models. See the supplemental information for results from the Reduced and uncorrelated models (Figs. S3, S4).

**Table 2:** Pairwise Pearson's correlation coefficient (*r*) between all pairs of models

	Full model	Reduced model	Uncorrelated model
Full models	–	0.97755	0.96645
Reduced model	0.98163	–	0.97344
Uncorrelated model	0.98546	0.98315	–

Pairwise correlation coefficients (*r*) for Black‐capped chickadee (*Poecile atricapillus*) models above diagonal and correlation coefficients for Carolina chickadee (*Poecile carolinensis*) models below diagonal.

The full models performed well for both species across the 10 cross‐validated runs, according to the AUC statistic (*P. atricapillus* AUC_mean_ = 0.843, AUC_SD_ = 0.007; *P. carolinensis* AUC_mean_ = 0.962, AUC_SD_ = 0.002). According to the criteria described by Araújo et al. (2005), predictive performance was “good” for *P. atricapillus* and “excellent” for *P. carolinensis* (Araújo et al. (2005), adapted from Swets (1988)). Because the AUC statistic is sensitive to the overall size of a species' distribution relative to the geographic study area (Phillips et al. 2006), the differences in AUC values reported here may reflect the range size differences between the two species, rather than any fundamental differences in predictive ability. The subset of climate variables with the highest relative contribution to construction of the full models differed slightly between the two species. For *P. atricapillus*, annual mean temperature contributed most to the model (79.2%), followed by maximum temperature of the warmest month (4.3%) and precipitation of the driest quarter (4.3%). For *P. carolinensis*, annual mean temperature also contributed most to the model (42.3%), followed by precipitation of the driest quarter (39.7%) and mean temperature of the warmest quarter (4.9%) (see Table S4 for percent contributions of all climate variables to the full model and Figure S5 for response curves for highest contributing climate variables).

**Figure 3:**
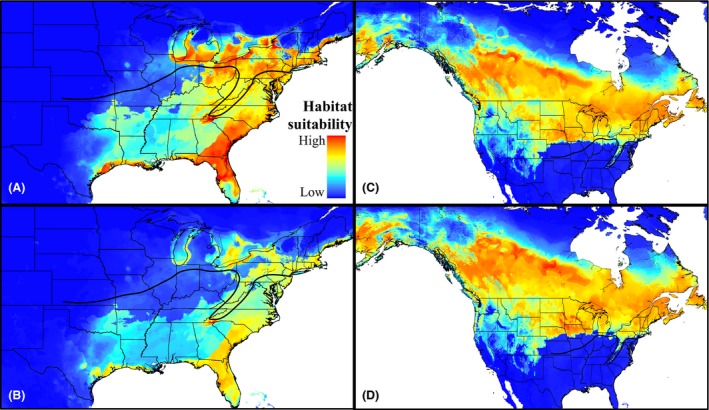
Year 2050 Climatically Suitable Areas for *Poecile carolinensis* and *Poecile atricapillus*. Potential distributions (full models) projected onto predicted climatic conditions for the year 2050, under the general circulation model HADGEM2‐ES. *Poecile carolinensis* climatically suitable areas for representative concentration pathways (RCPs) (A) 4.5 and (B) 8.5. *Poecile atricapillus* climatically suitable areas for RCPs (C) 4.5 and (D) 8.5.

When distribution models were projected onto future predicted climate scenarios for the year 2050, both *P. atricapillus* and *P. carolinensis* show a predicted northward shift in suitable habitat (Fig. 3). The predicted suitable habitat under both GCMs was similar (*r* > 0.87, Tables S2, S3), so we present here only the results from the HADGEM2‐ES circulation model (see Fig. S6 for CCSM4 results). Suitable habitat for *P. carolinensis* is predicted to drastically shift to the northeast under both representative concentration pathways (Fig. 3A and B). This predicted shift closely corresponds with locations where *P. carolinensis* have been reported to be moving north, in both Ohio (Bronson et al. 2003b) and Pennsylvania (Reudink et al. 2007; Taylor et al. 2014a). Both RCPs also show that higher elevation areas along the Appalachian Mountains will become climatically suitable for *P. carolinensis* in the years to come (Fig. 3A and B). The northward shift of suitable habitat for *P. atricapillus* is less drastic at the contact zone compared with *P. carolinensis*. However, under both RCPs, areas near the northern range edge of *P. atricapillus* in Canada and Alaska show a higher predicted climatic suitability by the year 2050 (Fig. 3C and D). Interestingly, future climate models predict a contraction in suitable habitat away from the hybrid zone for both species along the western half of the contact zone (Fig. 3).

**Figure 4:**
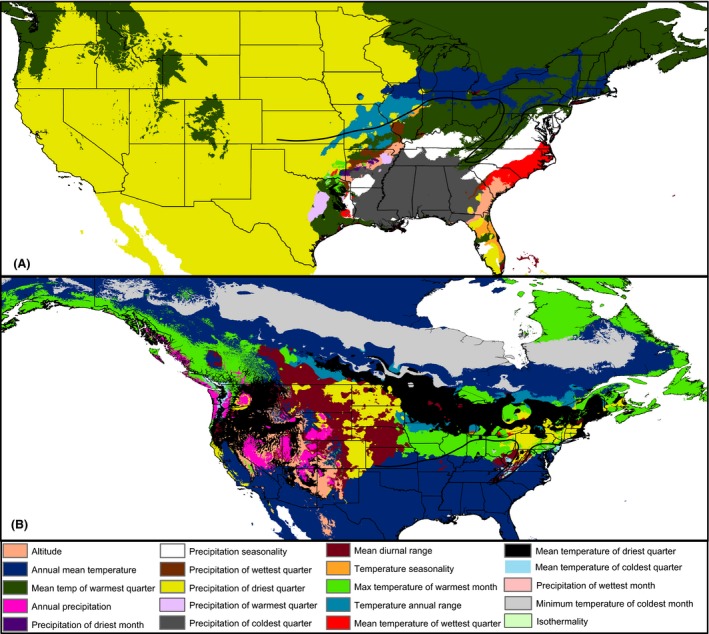
Limiting factor analysis. (A) Limiting factor analysis for *Poecile carolinensis*. Areas northeast of the hybrid zone limited primarily by annual mean temperature (dark blue) and mean temperature of the warmest quarter (dark green). Areas west of *P. carolinensis* limited primarily by precipitation of driest quarter (yellow). (B) Limiting factor analysis for *Poecile atricapillus*. Areas along much of the contact zone itself limited by maximum temperature of the warmest month (light green). Areas south of the hybrid zone limited primarily by annual mean temperature (dark blue). Northern edge of range limited by minimum temperature of coldest month (light gray). Note that some variables are only limiting for one species.

The limiting factor analysis indicated that throughout the current range of *P. carolinensis*, the limiting climate variable varies considerably (Fig. 4A). However, we were most interested in identifying the climate variables that, when changed, lead to the largest increase in the predicted probability of occurrence beyond the current species range limits. We found that at the eastern part of the contact zone, areas north of the zone are limited primarily by annual mean temperature (Bio1) and mean temperature of the warmest quarter (Bio10). The western range edge of *P. carolinensis* and areas north of the hybrid zone along the western part of the zone are limited primarily by precipitation of the driest quarter (Bio17) (Fig. 4A). The conclusion that temperature variables are limiting factors at areas northeast of the hybrid zone supports previous reports that warming temperatures were responsible for the northward shift of the hybrid zone over the last decade in these areas (Taylor et al. 2014a). The limiting factor analysis for *P. atricapillus* also indicated that the primary limiting climatic factor varies throughout the range (Fig. 4B). Along the northern range edge of *P. atricapillus* throughout much of Canada and Alaska, minimum temperature of the coldest month (Bio6) is a key limiting factor. Along much of the contact zone with *P. carolinensis*, the limiting factor is maximum temperature of the warmest month (Bio5). All areas south of the contact zone, outside the current range of *P. atricapillus,* are limited by annual mean temperature (Bio1). However, because abiotic factors were found to be less important for regulating range limits along the contact zone for *P. atricapillus*, these results should be taken with caution.

**Figure 5:**
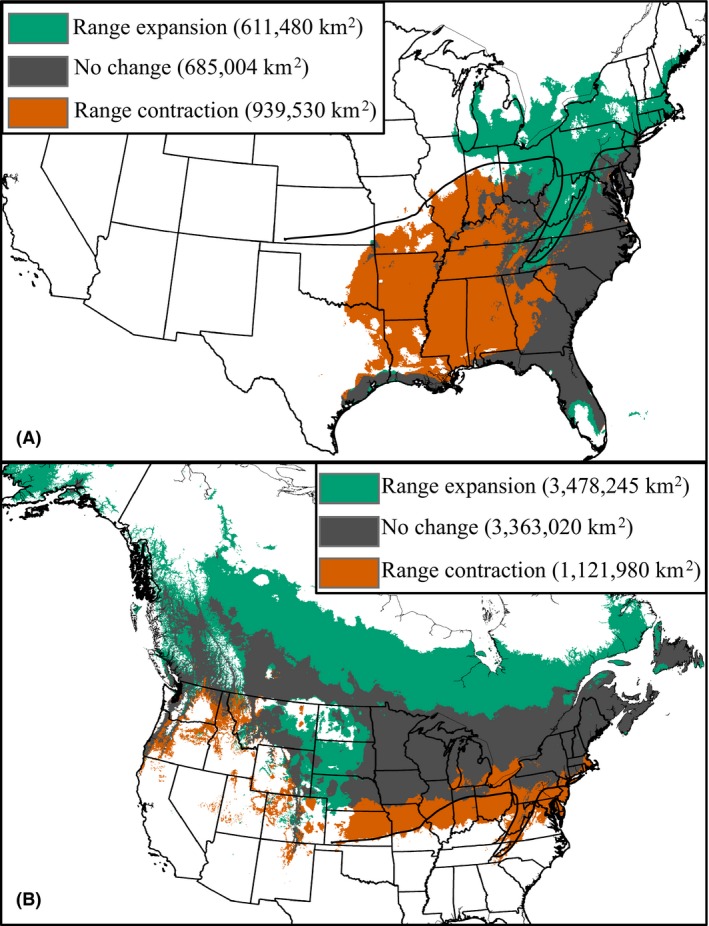
Range expansion/contraction under climate change. Area distribution changes between binary SDMs (species distribution modelings) for current and future (year 2050) climate conditions. (A) Change in binary SDMs for *Poecile carolinensis*. (B) Change in binary SDMs for *Poecile atricapillus*.

Our area analyses of potential range shifts for *P. carolinensis* under future climate conditions (year 2050) show large areas of both range expansion and range contraction (Fig. 5A). Areas of range expansion for *P. carolinensis* were identified in high elevation areas of the Appalachians, as well as areas northeast of the hybrid zone. Areas of future range contraction, on the other hand, were identified within the core of the range of *P. carolinensis,* as well as most areas south of the hybrid zone toward the western part of the range. The total area of suitable habitat for *P. carolinensis* is predicted to grow smaller under future climate change, as our analysis identified a larger area of predicted range contraction (939,530 km^2^) than range expansion (611,480 km^2^) (Fig. 5A). For *P. atricapillus*, range contraction along the hybrid zone is also predicted under climate change, especially along the western half of the contact zone (Fig. 5B). Because our current models predict climatically suitable habitat extending beyond the contact zone along much of the eastern part of the zone, predicted range contractions in these areas are likely to be less drastic. In fact, most areas along the eastern part of the contact zone are predicted to still be climatically suitable for *P. atricapillus* by the year 2050. In contrast to *P. carolinensis,* the total predicted area of climatically suitable habitat for *P. atricapillus* is predicted to grow under future climate change, with a larger area of predicted range expansion (3,478,245 km^2^) than range contraction (1,121,908 km^2^) (Fig. 5B). Again, because range limits for *P. atricapillus* are regulated more by biotic than abiotic factors along the contact zone, these results should be interpreted with caution.

## Discussion

Identifying the factors that shape species distribution patterns is a fundamental goal in the fields of ecology, biogeography, and evolution. Species distribution models can provide insight into the relative importance of the abiotic and biotic factors in shaping distributions. Here, we show that at a contact zone between the two hybridizing passerines, interspecific interactions play a larger role in shaping range limits in one species (*P. atricapillus*), while climate is more important for determining the range limits of the other (*P. carolinensis*). In addition, suitable habitat for *P. carolinensis* is projected to shift drastically northeast under both optimistic and pessimistic climate change scenarios, leading to a likely increase in the intensity and frequency of interspecific interactions with *P. atricapillus* in these areas. Thus, our results indicate that climate change has the potential to affect species distributions both directly (as for *P. carolinensis*) and indirectly (as for *P. atricapillus*). These results have broad implications for the dynamics of hybrid zones in general, as well as for understanding the complex ways in which natural populations will respond to climate change.

### Asymmetric effects of abiotic and biotic factors in shaping distributions between two chickadee species

Our modeling results show that the current distribution of *P. carolinensis* is shaped mainly by abiotic (climatic) factors. This is supported by the close correspondence between the species' potential and realized distributions (Fig. 2B). Conversely, distribution patterns for *P. atricapillus*, especially at the species' southern range edge at the contact zone with *P. carolinensis*, seem to be shaped primarily by biotic factors (interspecific interactions). The potential distribution of *P. atricapillus* extends south beyond the hybrid zone, and into the actual range of *P. carolinensis* (Fig. 2A). In other words, there is a large area of climatically suitable habitat extending south into areas not actually occupied by *P. atricapillus*. This mismatch between the potential and realized distribution for *P. atricapillus* supports the hypothesis that interspecific interactions are more important for shaping distribution patterns in this species along the hybrid zone.

In a hybrid zone where intrinsic selection against hybrids is strong, one might expect the potential, climatically suitable niche of both parental species to extend beyond the contact zone between them, into areas occupied by the other species. Under this scenario, the production of unfit hybrids within the hybrid zone would limit dispersal into otherwise climatically suitable environments. Because strong intrinsic selection acts against hybrid chickadees, in the form of reduced hatching success (Bronson et al. 2003b, 2005), we expected to find this pattern at the chickadee hybrid zone. Yet, interestingly, our results here are consistent with dispersal south by *P. atricapillus* being limited by interspecific hybridization, while the northward dispersal of *P. carolinensis* is limited mainly by climatic factors (Figs. 2, 4). This apparent asymmetry in the contributions of abiotic and biotic factors in regulating distribution patterns and the hybrid zone's location in these two species can be explained by a number of mechanisms.

First, it has been suggested that the northern range boundaries of many North American passerine birds are determined by metabolic performance and ability. Root (1988a,b) found that of 148 North American bird species examined, 60% have northern range boundaries that correspond with minimum daily January temperature isotherms. Root (1988a,b) concluded that winter temperatures limit these species because of the high energetic cost required to adjust metabolic rates to compensate for colder conditions. Further, physiological experiments in chickadees show that the basal metabolic rate of *P. carolinensis* is significantly lower than *P. atricapillus* after correcting for body mass differences and that *P. carolinensis* is less suited to colder environments than *P. atricapillus* (Olson et al. 2010). Thus, the chickadee hybrid zone may represent the northernmost thermal limit for *P. carolinensis*. Our distribution modeling output supports these hypotheses by two lines of evidence: Climatically suitable habitat for *P. carolinensis* does not extend north beyond the hybrid zone (Fig. 2B), and temperature variables are key limiting factors for *P. carolinensis* north of the hybrid zone (Fig. 4).

Second, *P. carolinensis* males appear to be socially dominant over *P. atricapillus* males (Bronson et al. 2003a). Dominance status in chickadees is associated with enhanced winter survival (Lemmon et al. 1997), as well as access to higher‐quality breeding territories (Smith 1991; Otter et al. 1999). Thus, dominant *P. carolinensis* males may competitively exclude *P. atricapillus* males at the parapatric range boundary. Mate choice preferences could also regulate the southern range limit in *P. atricapillus*. In captive settings, females of both chickadee species associate preferentially with dominant *P. carolinensis* males (Bronson et al. 2003a). Whether or not this female preference translates to asymmetric hybridization in the field remains an open question. However, *P. atricapillus* may be limited from dispersing southward if this species experiences a higher fitness cost to hybridization. For example, in the hybridizing Japanese freshwater minnows *Pseudorasbora pumila* and *P. parva*, all naturally occurring hybrids are of the F1 generation and are sterile. Additionally, these F1 hybrids all have *P. pumila* mtDNA, suggesting hybrids are only formed through the mating of *P. pumila* females with *P. parva* males. Because of strong selection against hybrids, it appears that *P. pumila* females waste considerably more reproductive effort than *P. parva* males, therefore suffering a higher fitness cost to hybridization (Konishi and Takata 2004). Although selection against hybrids appears to be similarly strong in chickadees (Taylor et al. 2014b), the possibility of such asymmetric fitness effects has not been explicitly tested. Additionally, *P. atricapillus* may be more likely to engage in heterospecific matings than *P. carolinensis* due to the reasons explained above*,* which could result in greater fitness costs for *P. atricapillus* even if, on a case‐by‐case basis, the consequences of hybridization are identical for the two species. Such a process could lead to demographic shifts in favor of *P. carolinensis* at the parapatric range edge.

In addition to identifying the factors that regulate range limits along a natural hybrid zone, our modeling efforts also provide insight into some longstanding macroecological questions. For example, a central hypothesis regarding the factors regulating species distributions posits that biotic factors will be more important at a species' equatorial (low latitude) range limit, while abiotic factors will be more important at the poleward (high latitude) limit (MacArthur 1972; Schemske et al. 2009; Hargreaves et al. 2013). While this hypothesis has gained considerable empirical support across a variety of taxonomic groups (reviewed in Schemske et al. 2009), further research is needed to determine how widespread this macroecological pattern is. For instance, a recent study that examined 214 North American amphibian and reptile species found that this hypothesis holds true for reptiles, but not for amphibians (Cunningham et al. 2015). In a similar fashion, a recent review of 105 studies examining a total of 178 terrestrial and aquatic species found that abiotic factors are supported more often than biotic factors in delimiting equatorial range boundaries (Cahill et al. 2014). Despite these disagreements, our results lend support to the hypothesis that biotic factors regulate equatorial range limits, as the predicted southern range limit for *P. atricapillus* extends beyond the realized range boundary. Our results also show that for these two species, abiotic climatic factors are more important for delimiting poleward range limits. The northern range limit for *P. carolinensis* closely aligns with that predicted by distribution models. For *P. atricapillus,* the northern range boundary is not as accurately predicted by our models, which could be due to the fact the majority of our occurrence points are from the range core. However, our limiting factor analysis for *P. atricapillus* indicates that minimum temperature of the coldest month is a key limiting factor at the northern range boundary (Fig. 4B), which supports previous conclusions that minimum winter temperatures limit the northern range limits of many North American bird species (Root 1988b). Further work should examine the frequencies at which equatorial and poleward range boundaries correspond with the position of hybrid zones.

Another potential factor that might explain the discrepancy between the potential and realized distribution along the contact zone is the fact that we used distribution data from western North America to infer potential distributions in the eastern part of the range, particularly for *P. atricapillus*. Because of the large area occupied by *P. atricapillus*, and the history of climatic fluctuations in North America since the last glacial maximum (Hewitt 2004), the eastern and western populations may not be ecologically equivalent. Further, a recent study examining signatures of genetic structure in *P. atricapillus* using samples from across the species' range found that populations in the western part of the range are significantly structured, while eastern populations are not (Adams and Burg 2015). This genetic structure was attributed to the presence of major mountain ranges, including the Cascade and Rocky Mountains. However, when we used only occurrences from the eastern portion of *P. atricapillus's* range to construct distribution models, the model overprediction along the hybrid zone was still apparent (data not shown). Thus, we conclude that the discrepancy between the potential and realized range boundary is not due to the inclusion of western occurrences for this species.

Species distribution modeling has been used in a small, yet growing number of studies aimed at examining hybrid zone dynamics. Swenson (2006) used an SDM framework to study four north‐to‐south avian hybrid zones that cluster at the North American Great Plains suture zone. He found a large mismatch between the potential and realized distribution for all four parental species occupying the western half of the United States, but not for the four parental species located to the east. He concluded that the four eastern species' ranges are limited by climatic factors, whereas biotic interactions likely limit range boundaries for the western species (Swenson 2006). However, unlike the chickadee hybrid zone described here, the Great Plains suture zone appears stable in space and time. More recently, Engler et al. (2013) used a MAXENT modeling approach to study a European hybrid zone between Melodious (*Hippolais polyglotta*) and Icterine (*H. icterina*) warblers that appears to be moving, possibly as a result of climate change. They found that for both species, potential distributions extended far beyond the hybrid zone and into the range of the other species. Thus, biotic interactions are more important for determining the breeding ranges of these two migratory species, and these results support the hypothesis that the warbler hybrid zone is a tension zone (Engler et al. 2013). The chickadee hybrid zone described here is also hypothesized to be a moving tension zone (Bronson et al. 2005), yet is unique to the warbler study in that a mismatch between the potential and realized distribution is only apparent in one species.

When teasing apart the relative importance of abiotic and biotic factors in driving range boundaries, it is important to note that biotic interactions themselves can be climate dependent. For example, the intensity of competition between two stream salmonid fish species was found to depend on temperature gradients (Taniguchi and Nakano 2000). The degree to which reproductive interactions between species depend on climate, on the other hand, is less well characterized (Chunco 2014). In chickadees, further field studies are needed to assess the cumulative effects of interspecific interactions, and whether these interactions are themselves affected by climate.

### Predicted hybrid zone movement under climate change

The chickadee hybrid zone is moving rapidly northward (Bronson et al. 2003a; Reudink et al. 2007; Taylor et al. 2014a). *Poecile carolinensis* is expanding its range northward and displacing *P. atricapillus* at a rate of about 10 km/decade (Taylor et al. 2014a). This latitudinal range shift is now a diagnostic feature of populations responding to the effects of climate warming (Chen et al. 2011). Indeed, a recent study by Taylor et al. (2014a) found that climate warming in the recent past has played an important role in the contact zone's northward shift in Pennsylvania over the last decade. Specifically, it was found that the northern extent of *P. carolinensis* is currently associated with average minimum daily winter temperatures. Using temperature data as well as species occurrence records, Taylor et al. were able to accurately hindcast the location of the hybrid zone a decade ago. As temperatures warmed, the hybrid zone shifted north. While the Taylor et al.'s study is informative regarding current and past hybrid zone dynamics at the eastern portion of the zone, questions about zone movement to the west and whether the zone will continue to shift north in the future remained unanswered. Barring rapid adaptation to the changing climate, our distribution modeling output suggests that northward movement of the hybrid zone is likely to continue into the future, as suitable habitat for *P. carolinensis* is predicted to shift drastically northeast under multiple projected climate change scenarios for the year 2050 (Figs. 3, 5, S6).

However, our study also highlights the fact that hybrid zone movement in chickadees might be more geographically variable than previously thought. Our models predict a northeast shift in suitable habitat for *P. carolinensis*, corresponding to observed hybrid zone movements. Our models also predict that habitat will become unsuitable for *P. carolinensis* at areas south of the contact zone to the west, particularly in the states of Illinois and Missouri (Fig. 5). To our knowledge, hybrid zone movement has not been reported in these areas (Enstrom and Bollinger 2009), and future work should focus on hybrid zone dynamics in these areas.

Our modeling results suggest that the southern range edge of *P. atricapillus* is determined more by biotic than climatic factors. Thus, distribution patterns in this species, at least at the southern edge, are less likely to be directly influenced by a changing climate (Fig. 3). However, our results also show that large portions of the current range of *P. atricapillus* will become climatically suitable for *P. carolinensis* by the year 2050, which may result in intensified interspecific interactions between these two species, especially along the eastern half of the contact zone. Thus, even though climate change is expected to have minimal direct effects on the range of *P. atricapillus*, the climate‐mediated expansion of *P. carolinensis* may cause *P. atricapillus* to retreat northward. Together, our modeling results for both chickadee species highlight the ways climate change can influence species distributions through direct as well as indirect effects.

Although the direct effects of climate change on species distributions are relatively well understood (Parmesan 2006), indirect effects are less well characterized. Climate change can indirectly affect species boundaries by altering interspecific interactions with predators, competitors, or parasites (Thomas 2010). However, studies demonstrating a climate change‐induced range expansion in one species that indirectly affects the range boundary of a second, interacting species, are rare. This phenomenon will likely become more frequent as climate‐mediated range shifts bring previously isolated populations into contact.

It is important to note that our distribution models exclusively include climate variables, and there may be other aspects of the environment (e.g., vegetation, tree species available for nesting) important for determining chickadee distributions that are missed by our modeling efforts. However, both chickadee species examined here are widespread generalists that occupy a variety of forest habitats across the United States and Canada. Within these habitats, chickadees excavate nesting cavities in dead or decaying trees, and to our knowledge, no differences exist in the tree species selected for nesting between the two species (Albano 1992; Martin et al. 2004). Therefore, any climate change‐induced distribution shift in the underlying vegetation is less likely to have an indirect effect on chickadees than it would a more specialized species or a species with narrow nesting requirements. Additionally, a large proportion of the occurrence data used in our models derive from the citizen science database eBird (Sullivan et al. 2009). Such databases are increasingly being used to ask questions that otherwise would not be accessible or feasible (Silvertown 2009). Indeed, data from eBird have been widely utilized in the scientific literature, with over 90 peer‐reviewed publications making use of the database in the last decade (Sullivan et al. 2014). Despite growing enthusiasm for citizen science applications, the adoption of these types of data raises questions concerning data accuracy and quality control. In the case of eBird, data are filtered by local experts and unusual records are flagged for further inspection. However, the possibility exists that some records are incorrectly entered or individuals are misidentified, which could lead to biased conclusions about distribution patterns. For morphologically similar species, such as chickadees, these issues are even more important to consider. However, given the thorough data‐filtering methods we have employed (see Methods), and the wide geographic extent from which our occurrence records are drawn, it is unlikely that these pitfalls have affected our results.

Moving hybrid zones are now recognized to be common in nature (Buggs 2007). Although hybrid zones can move for reasons other than a changing climate, several good examples link climate change with zone movement (Britch et al. 2001; Scriber 2011; Taylor et al. 2014a). Indeed, moving hybrid zones may represent sensitive indicators for anthropogenic climate change (Taylor et al. 2015). Further, the effects of climate change on hybridizing taxa will have widespread evolutionary consequences. As species shift geographically due to climate change, hybridization between previously isolated groups is predicted to become more common (Chunco 2014). Climate change will cause new hybrid zones to form (e.g., Garroway et al. 2010), and in some cases, the extent of hybridization between currently sympatric taxa will increase (e.g., Gérard et al. 2006). In this way, hybridization might result in strengthened reproductive barriers between populations, finalizing the speciation process through reinforcement (Servedio and Noor 2003). On the other hand, hybridization can weaken reproductive isolating barriers, resulting in species fusion (Taylor et al. 2006). Finally, hybridization can act as an evolutionary stimulus by promoting the introgression of adaptive genetic material across species boundaries (Abbott et al. 2013).

For chickadees, some of these evolutionary outcomes are more likely than others. Based on divergence in mitochondrial DNA, these two sister species likely diverged as far back as 4 million years ago (Price 2008; Harr and Price 2014; Harris et al. 2013). Now in secondary contact, reproductive isolation between the two species appears significant. Recent genomic work examining a transect of the chickadee hybrid zone in Pennsylvania identified very few, if any, early‐generation backcross hybrids (Taylor et al. 2014a). This result, in combination with the fact that most loci throughout the genome display narrow cline widths (Taylor et al. 2014b), suggests hybrid chickadees face strong intrinsic selection pressure and genetic introgression is rare. Although genetic introgression across species boundaries appears to be at low levels currently, our models predict increased opportunities for interspecific hybridization under climate change, especially at the eastern half of the contact zone where *P. carolinensis* is shifting north into the range of *P. atricapillus*. These dynamics may increase the likelihood and frequency of genetic introgression across species boundaries, especially if some of the introgressing genes offer adaptive benefits in rapidly changing climatic conditions.

In order to persist in the face of rapid climate change, species must shift their distributions to more favorable climates, respond through phenotypic plasticity, or adapt. Whether or not evolutionary adaptation to changing environments can keep up with the pace of climate change remains an open question for many species (Hoffmann and Sgrò 2011). Predictions concerning evolutionary adaptation are complicated by the fact that species exist within a web of coevolutionary interactions, which themselves can be altered due to climate change (Northfield and Ives 2013). However, interspecific hybridization can facilitate adaptive potential by introducing genetic variation (Abbott et al. 2013). Indeed, theoretical work suggests that introgressive hybridization can rescue an extinction‐prone species after an abrupt environmental change (Baskett and Gomulkiewicz 2011). For example, in Darwin's Finches, hybridization has led to increased standing genetic variation, which has facilitated adaptation to changing environmental conditions (Grant and Grant 2010). With chickadees, such a mechanism is more likely along the eastern half of the contact zone, where hybridization frequency is expected to increase under climate change. In contrast, climatically suitable habitat for both species is expected to retreat away from the contact zone along its western half, potentially reducing the rate of interspecific hybridization. If chickadee populations along the western part of the contact zone cannot adequately track these changes in climate through dispersal, their persistence will rely on their capacity for local adaptation or phenotypic plasticity. Although SDM methods cannot explicitly test for future potential for local adaptation, such predictive efforts are useful in identifying areas for future study where evolutionary adaptation may be important for species persistence.

In sum, our study demonstrates the relative importance of biotic and abiotic factors in determining range limits for two parapatrically distributed, hybridizing bird species and highlights the insights that can be gained from using species distribution models. We also show that for widespread species that share a very long zone of contact, different climatic mechanisms may be at work at different parts of the zone, which can directly and indirectly influence competitive as well as reproductive interactions between species. These implications are important to consider for the study of dynamic, moving hybrid zones, as well as for understanding the complex ways that natural populations will respond to a rapidly changing global climate.

## Conflict of Interest

None declared.

## Supporting information


**Figure S1.** Binary SDMs for *P. carolinensis* and *P. atricapillus*.

**Figure S2**. Species distribution models (full models) zoomed in on hybrid zone.

**Figure S3**. MAXENT species distribution models (reduced models) for *P. atricapillus* and *P. carolinensis* under current conditions.

**Figure S4**. MAXENT species distribution models (uncorrelated models) for *P. atricapillus* and *P. carolinensis* under current conditions.

**Figure S5**. MAXENT response curves for highest contributing climate variables.

**Figure S6**. Future Climatically Suitable Areas for *P. carolinensis* and *P. atricapillus*.

**Table S1.** Pairwise Pearson's correlation coefficient (r) matrix for climate variables.
**Table S2**. Pairwise Pearson's Correlation coefficient (r) matrix for future projected climate models – *Poecile atricapillus*.
**Table S3**. Pairwise Pearson's Correlation coefficient (r) matrix for future projected climate models – *Poecile carolinensis*.
**Table S4.** Climate variable percent contributions to the full model for each chickadee species.
