# Supplementary figures and images for: Differential effects of climate and species interactions on range limits at a hybrid zone: potential direct and indirect impacts of climate change
Source: Ecol Evol. 2015 Oct 19;5(21):5120–37. doi: 10.1002/ece3.1774 (PMC4662315; doi:10.1002/ece3.1774)

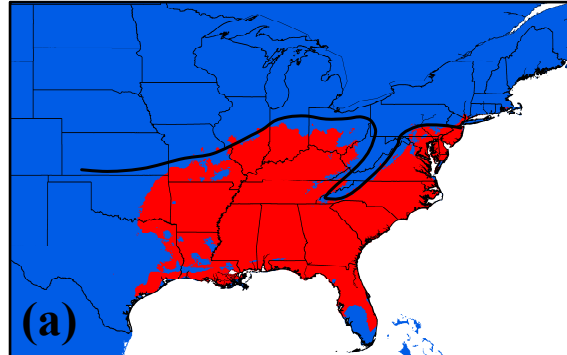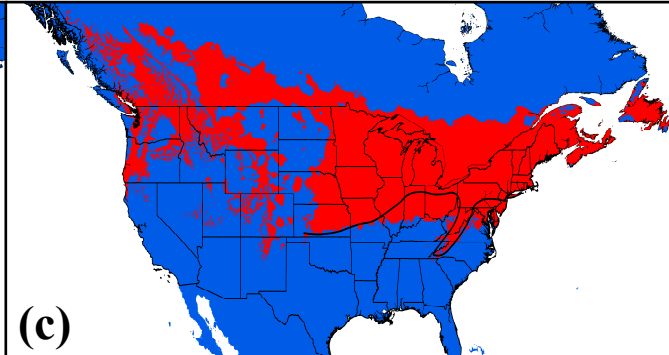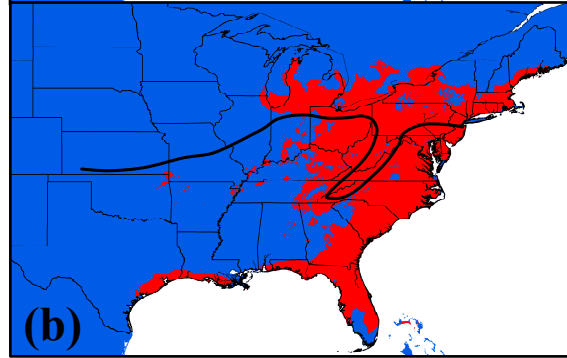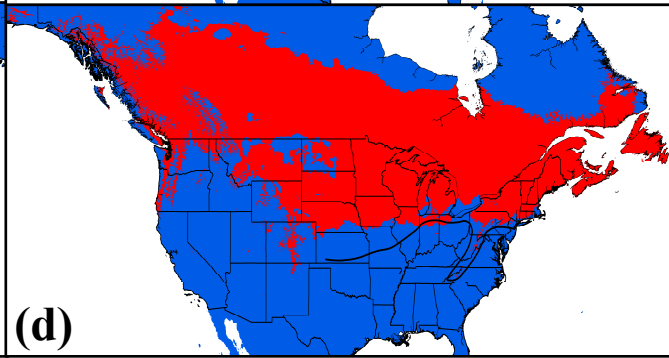

Supplement: Supplementary file 1 — Figure S1. Binary SDMs for P. carolinensis and P. atricapillus. [file ECE3-5-5120-s001.pdf]

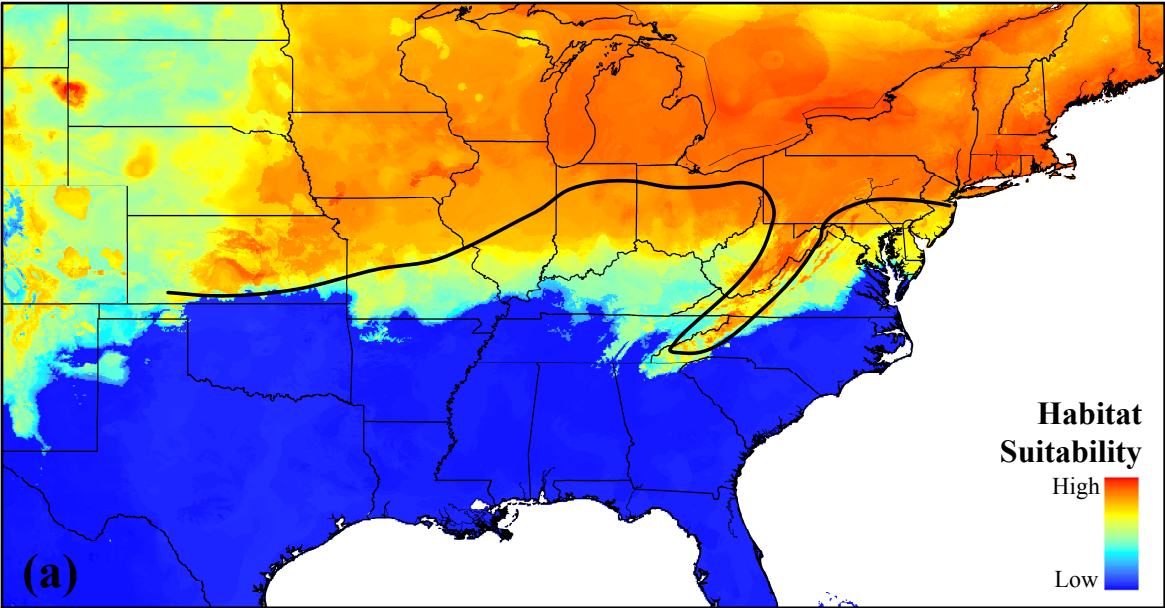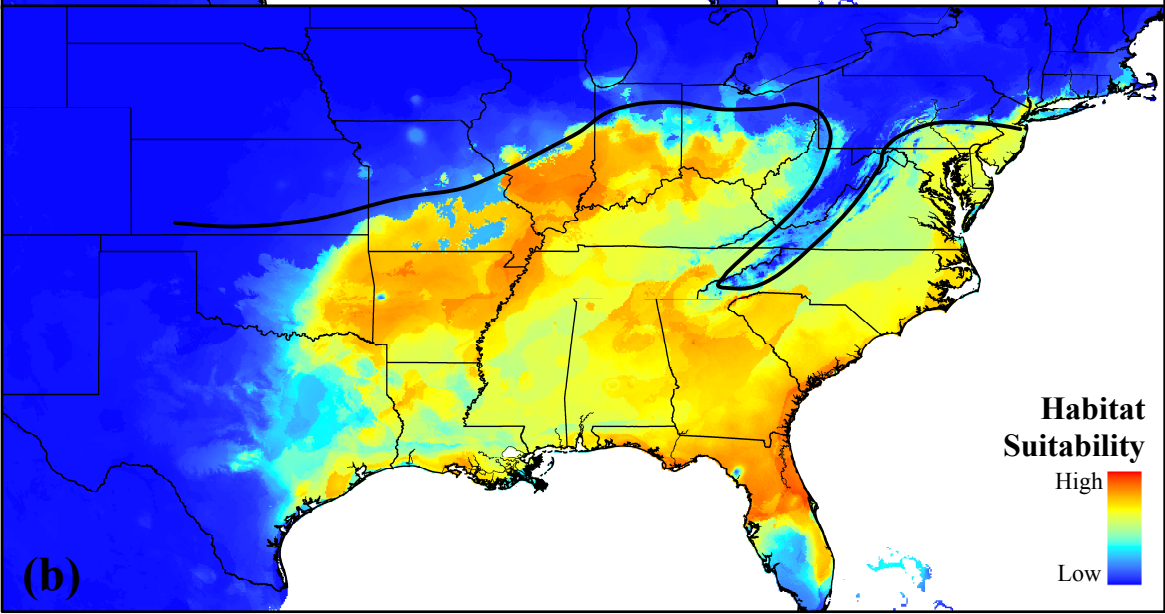

Supplement: Supplementary file 2 — Figure S2. Species distribution models (full models) zoomed in on hybrid zone. [file ECE3-5-5120-s002.pdf]

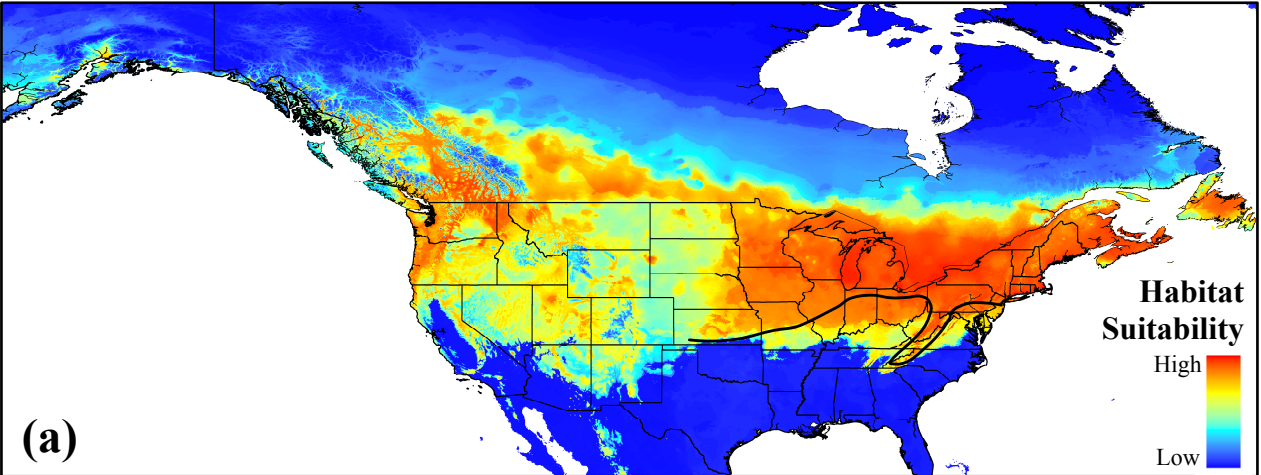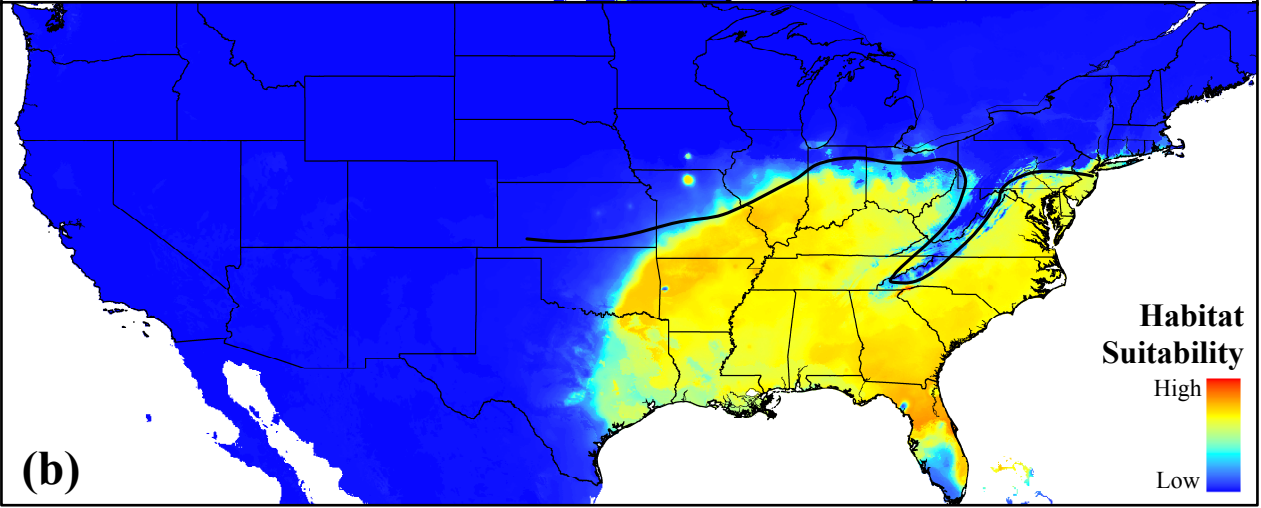

Supplement: Supplementary file 3 — Figure S3. MAXENT species distribution models (reduced models) for P. atricapillus and P. carolinensis under current conditions. [file ECE3-5-5120-s003.pdf]

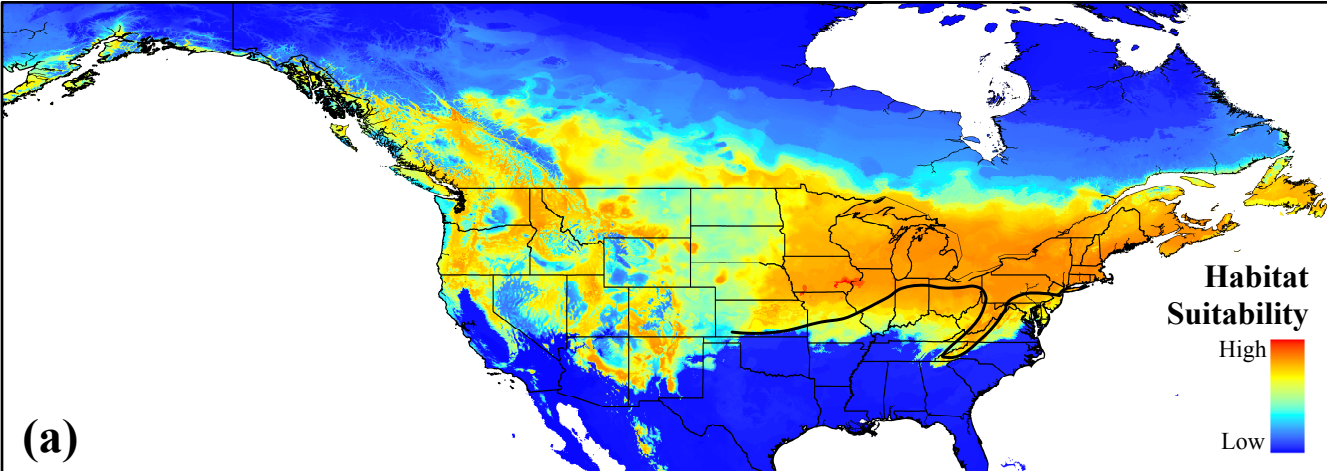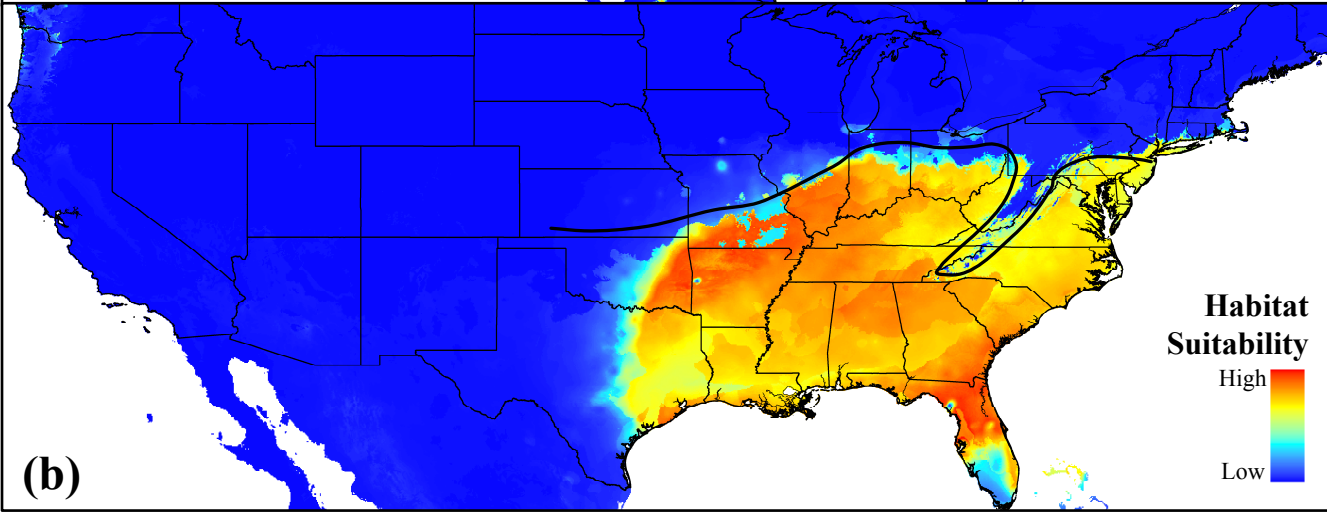

Supplement: Supplementary file 4 — Figure S4. MAXENT species distribution models (uncorrelated models) for P. atricapillus and P. carolinensis under current conditions. [file ECE3-5-5120-s004.pdf]

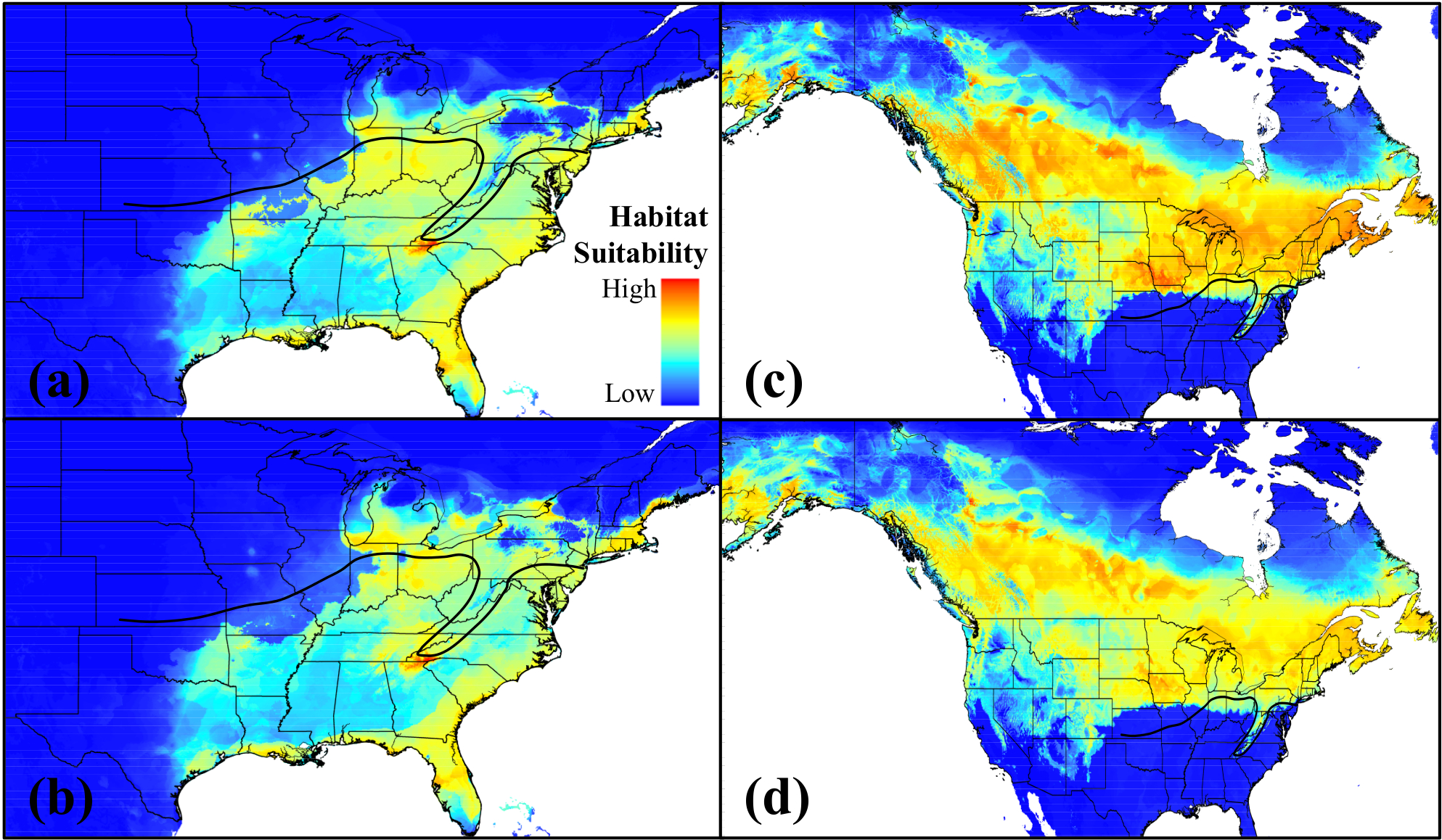

Supplement: Supplementary file 6 — Figure S6. Future Climatically Suitable Areas for P. carolinensis and P. atricapillus. [file ECE3-5-5120-s006.jpg]
