# Supplementary material for: Differential effects of climate and species interactions on range limits at a hybrid zone: potential direct and indirect impacts of climate change
Source: Ecol Evol. 2015 Oct 19;5(21):5120–37. doi: 10.1002/ece3.1774 (PMC4662315; doi:10.1002/ece3.1774)

Response of *Poecile atricapillus* to Annual Mean Temp. (Bio1)

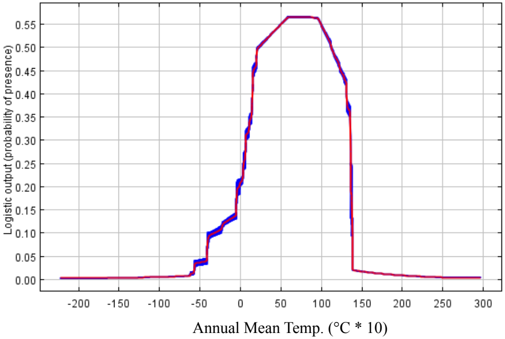

Response of *Poecile carolinensis* to Annual Mean Temp. (Bio1)

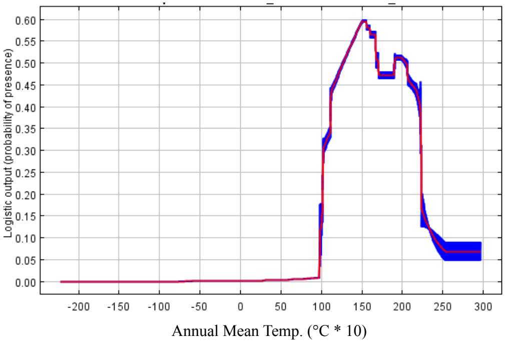

Response of *Poecile atricapillus* to Max. Temp. of Warmest Month (Bio5)

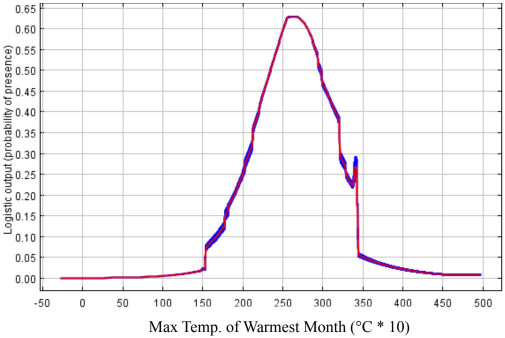

Response of *Poecile carolinensis* to Precipitation of Driest Quarter (Bio17)

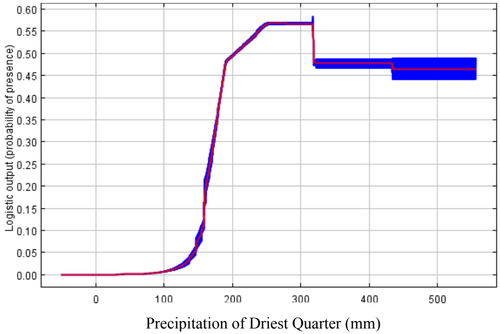

Response of *Poecile atricapillus* to Precipitation of Driest Quarter (Bio17)

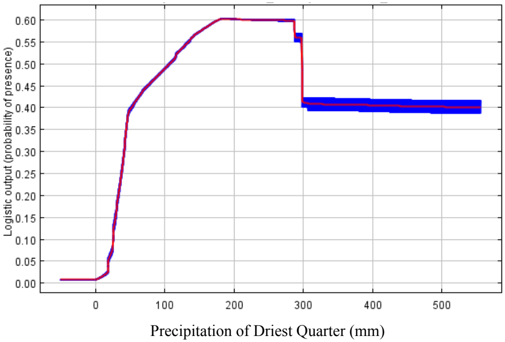

Response of *Poecile carolinensis* to Mean Temp. of Warmest Quarter (Bio10)

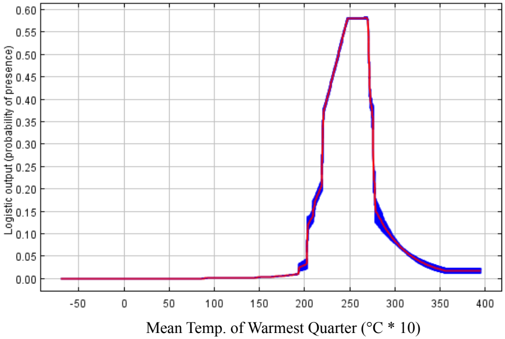

Supplement: Supplementary file 5 — Figure S5. MAXENT response curves for highest contributing climate variables. [file ECE3-5-5120-s005.pdf]
