# Supplementary material for: Differential effects of climate and species interactions on range limits at a hybrid zone: potential direct and indirect impacts of climate change
Source: Ecol Evol. 2015 Oct 19;5(21):5120–37. doi: 10.1002/ece3.1774 (PMC4662315; doi:10.1002/ece3.1774)
Supplement: Supplementary file 7 — Table S1. Pairwise Pearson's correlation coefficient (r) matrix for climate variables. Table S2. Pairwise Pearson's Correlation coefficient (r) matrix for future projected climate models – Poecile atricapillus. Table S3. Pairwise Pearson's Correlation coefficient (r) matrix for future projected climate models – Poecile carolinensis. Table S4. Climate variable percent contributions to the full model for each chickadee species. [file ECE3-5-5120-s007.docx]

**Supporting Information Figure Legends**

**Figure S1** – Binary SDMs for *P. carolinensis* and *P. atricapillus*. Full models generated using the ‘Maximum training sensitivity plus specificity’ threshold as specified in MAXENT. **(a)** Threshold applied to *P. carolinensis* full model under contemporary conditions. **(b)** Threshold applied to *P. carolinensis* full model for year 2050 conditions – GCM HADGEM2-ES; RCP 4.5. **(c)** Threshold applied to *P. atricapillus* full model under contemporary conditions. **(d)** Threshold applied to *P. atricapillus* full model for year 2050 conditions. Red areas = Suitable habitat. Blue areas = Unsuitable habitat.

**Figure S2** - Species distribution models (Full Models) zoomed in on hybrid zone. **(a)** The potential distribution for Black-capped (*P. atricapillus*) chickadees extends south beyond the hybrid zone, into the actual range of the Carolina chickadee. **(b)** The potential distribution for the Carolina (*P. carolinensis*) chickadee does not extend beyond the contact zone. Approximate location of hybrid zone drawn as heavy black line (based on Taylor *et al*., 2014a).

**Figure S3** - MAXENT species distribution models (Reduced Models) for *P. atricapillus* and *P. carolinensis* under current conditions. **(a)** Black-capped (*P. atricapillus*) chickadee potential distribution – Reduced Model. **(b)** Carolina (*P. carolinensis*) chickadee potential distribution – Reduced Model. Warmer colors indicate higher predicted habitat suitability. Approximate location of hybrid zone drawn as heavy black line (based on Taylor *et al.*, 2014a).

**Figure S4** – MAXENT species distribution models (Uncorrelated Models) for *P. atricapillus* and *P. carolinensis* under current conditions. **(a)** Black-capped (*P. atricapillus*) chickadee potential distribution – Uncorrelated Model. **(b)** Carolina (*P*. carolinensis) chickadee potential distribution – Uncorrelated Model. Warmer colors indicate higher predicted habitat suitability. Approximate location of hybrid zone drawn as heavy black line (based on Taylor *et al.*, 2014a).

**Figure S5** – MAXENT response curves for highest contributing climate variables. The response curves for the top three contributing variables to construction of the full models for both species. Response curves show how the logistic output changes along each corresponding climate axis. Response curves were generated by running a MAXENT model using only the corresponding variable. Red lines indicate the mean response of the 10 replicated runs, while blue lines show +/- one standard deviation.

**Figure S6** - Future Climatically Suitable Areas for *P. carolinensis* and *P. atricapillus*. Potential distributions (Full Models) projected onto predicted climatic conditions for the year 2050, under the general circulation model CCSM4. *Poecile carolinensis* models for Representative Concentration Pathways (RCPs) **(a)** 4.5, and **(b)** 8.5. *Poecile atricapillus* models for RCPs **(c)** 4.5, and **(d)** 8.5.

|  | **Bio1** | **Bio2** | **Bio3** | **Bio4** | **Bio5** | **Bio6** | **Bio7** | **Bio8** | **Bio9** | **Bio10** | **Bio11** | **Bio12** | **Bio13** | **Bio14** | **Bio15** | **Bio16** | **Bio17** | **Bio18** | **Bio19** |
| --- | --- | --- | --- | --- | --- | --- | --- | --- | --- | --- | --- | --- | --- | --- | --- | --- | --- | --- | --- |
| Bio1 | 1.0000 |  |  |  |  |  |  |  |  |  |  |  |  |  |  |  |  |  |  |
| Bio2 | **0.7050** | 1.0000 |  |  |  |  |  |  |  |  |  |  |  |  |  |  |  |  |  |
| Bio3 | **0.8713** | **0.8097** | 1.0000 |  |  |  |  |  |  |  |  |  |  |  |  |  |  |  |  |
| Bio4 | **-0.8118** | -0.4986 | **-0.8747** | 1.0000 |  |  |  |  |  |  |  |  |  |  |  |  |  |  |  |
| Bio5 | **0.9309** | **0.8267** | **0.7881** | -0.5874 | 1.0000 |  |  |  |  |  |  |  |  |  |  |  |  |  |  |
| Bio6 | **0.9594** | 0.6009 | **0.8947** | **-0.9293** | **0.8146** | 1.0000 |  |  |  |  |  |  |  |  |  |  |  |  |  |
| Bio7 | -0.6435 | -0.1646 | -0.6784 | **0.9311** | -0.3412 | **-0.8232** | 1.0000 |  |  |  |  |  |  |  |  |  |  |  |  |
| Bio8 | 0.6097 | 0.4772 | 0.4063 | -0.2029 | 0.6732 | 0.4432 | -0.0593 | 1.0000 |  |  |  |  |  |  |  |  |  |  |  |
| Bio9 | **0.8892** | 0.6415 | **0.8733** | **-0.8727** | **0.7765** | **0.9255** | **-0.7397** | 0.3000 | 1.0000 |  |  |  |  |  |  |  |  |  |  |
| Bio10 | **0.9521** | **0.7176** | **0.7499** | -0.5983 | **0.9794** | **0.8406** | -0.4036 | **0.7243** | **0.7731** | 1.0000 |  |  |  |  |  |  |  |  |  |
| Bio11 | **0.9785** | 0.6675 | **0.9155** | **-0.9117** | **0.8564** | **0.9941** | **-0.7728** | 0.4945 | **0.9247** | **0.8742** | 1.0000 |  |  |  |  |  |  |  |  |
| Bio12 | 0.3928 | -0.0841 | 0.2165 | -0.4539 | 0.1987 | 0.4457 | -0.5279 | 0.0483 | 0.4150 | 0.2876 | 0.4154 | 1.0000 |  |  |  |  |  |  |  |
| Bio13 | 0.3928 | -0.0307 | 0.3018 | -0.4824 | 0.1937 | 0.4547 | -0.5473 | 0.1032 | 0.4099 | 0.2727 | 0.4281 | **0.9025** | 1.0000 |  |  |  |  |  |  |
| Bio14 | 0.2997 | -0.1332 | 0.0829 | -0.3297 | 0.1374 | 0.3357 | -0.4096 | -0.0052 | 0.3334 | 0.2274 | 0.3087 | **0.8634** | 0.6079 | 1.0000 |  |  |  |  |  |
| Bio15 | -0.0923 | 0.1176 | 0.1102 | 0.1003 | -0.0403 | -0.0994 | 0.1216 | 0.1591 | -0.1466 | -0.0644 | -0.0831 | -0.5092 | -0.1460 | **-0.7492** | 1.0000 |  |  |  |  |
| Bio16 | 0.3718 | -0.0492 | 0.2759 | -0.4652 | 0.1753 | 0.4336 | -0.5311 | 0.0730 | 0.3937 | 0.2519 | 0.4061 | **0.9251** | **0.9898** | 0.6333 | -0.1949 | 1.0000 |  |  |  |
| Bio17 | 0.3272 | -0.1175 | 0.1117 | -0.3590 | 0.1596 | 0.3655 | -0.4362 | 0.0063 | 0.3616 | 0.2498 | 0.3379 | **0.8907** | 0.6440 | **0.9930** | **-0.7447** | 0.6693 | 1.0000 |  |  |
| Bio18 | 0.3259 | -0.0376 | 0.1035 | -0.2270 | 0.1979 | 0.2750 | -0.2520 | 0.3629 | 0.1898 | 0.3013 | 0.2836 | **0.7482** | 0.6803 | **0.7089** | -0.3648 | 0.6855 | **0.7200** | 1.0000 |  |
| Bio19 | 0.3257 | -0.0711 | 0.2712 | -0.4969 | 0.1301 | 0.4370 | -0.5808 | -0.1723 | 0.4757 | 0.1830 | 0.3914 | **0.8959** | **0.8390** | **0.7108** | -0.4019 | **0.8685** | **0.7436** | 0.4196 | 1.0000 |

**Supporting Information Tables**

**Table S1 -** Pairwise Pearson’s correlation coefficient (r) matrix for climate variables

Pearson’s correlation coefficient (r) of all climate variables used for modeling. Values generated by selecting 10,000 points at random from the geographic study area, and extracting climate data from each point. Correlated variables with r>0.7 in bold.

**Table S2** – Pairwise Pearson’s Correlation coefficient (r) matrix for future projected climate models – *Poecile atricapillus*.

|  | HadGEM2-ES. RCP 4.5 | HadGEM2-ES. RCP 8.5 |
| --- | --- | --- |
| CCSM4. RCP 4.5 | 0.93900 | - |
| CCSM4. RCP 8.5 | - | 0.91325 |

**Table S3** – Pairwise Pearson’s Correlation coefficient (r) matrix for future projected climate models – *Poecile carolinensis.*

|  | HadGEM2-ES. RCP 4.5 | HadGEM2-ES. RCP 8.5 |
| --- | --- | --- |
| CCSM4. RCP 4.5 | 0.90592 | - |
| CCSM4. RCP 8.5 | - | 0.87443 |

**Table S4 – Climate variable percent contributions to the Full Model for each chickadee species.**

|  | *Poecile atricapillus* | *Poecile carolinensis* |
| --- | --- | --- |
| Annual Mean Temperature (Bio1) | 79.2 % | 42.3 % |
| Mean Diurnal Range in Temperature (Bio2) | 2.8 % | 0.8 % |
| Isothermality (Bio3) | 0.5 % | 0.1 % |
| Temperature Seasonality (Bio4) | 0.6 % | 0.7 % |
| Max Temperature of Warmest Month (Bio5) | 4.3 % | 0.7 % |
| Min Temperature of Coldest Month (Bio6) | 1.7 % | 0.6 % |
| Temperature Annual Range (Bio7) | 0.6 % | 1 % |
| Mean Temperature of Wettest Quarter (Bio8) | 0.1 % | 0.3 % |
| Mean Temperature of Driest Quarter (Bio9) | 0.3 % | 0.2 % |
| Mean Temperature of Warmest Quarter (Bio10) | 0.2 % | 4.9 % |
| Mean Temperature of Coldest Quarter (Bio11) | 2.5 % | 0.6 % |
| Annual Precipitation (Bio12) | 1.2 % | 3.9 % |
| Precipitation of Wettest Month (Bio13) | 0 % | 0.1 % |
| Precipitation of Driest Month (Bio14) | 0.4 % | 2.2 % |
| Precipitation Seasonality (Bio15) | 0.6 % | 0.6 % |
| Precipitation of Wettest Quarter (Bio16) | 0.1 % | 0.7 % |
| Precipitation of Driest Quarter (Bio17) | 3.9 % | 39.7 % |
| Precipitation of Warmest Quarter (Bio18) | 0.3 % | 0.5 % |
| Precipitation of Coldest Quarter (Bio19) | 0.1 % | 0.5 % |
| Altitude | 0.6 % | 0 % |
